# Descemet’s membrane folds in ochronosis: a case report

**DOI:** 10.1186/s13256-022-03599-x

**Published:** 2022-10-02

**Authors:** Otavio de Azevedo Magalhaes, Dunia Abdel Rahman Abu Hwas

**Affiliations:** 1 Department of Ophthalmology, Federal University of São Paulo (UNIFESP), São Paulo, Brazil; 2 Porto Alegre Eye’s Bank Hospital, 285 Walter Boehl Street, 333 Mostardeiro St., Office 503, Porto Alegre, Rio Grande do Sul Brazil

**Keywords:** Alkaptonuria, Ochronosis, Scleral stiffness, Descemet’s membrane folds, Case report

## Abstract

**Background:**

We present this report of a new ophthalmic finding in a patient with ochronosis.

**Case presentation:**

An 85-year-old Caucasian male patient with bilateral dark temporal and nasal pigmentation of conjunctiva and sclera was referred to our hospital owing to low visual acuity. On biomicroscopic examination, bilateral horizontal Descemet’s membrane folds were observed. Corneal tomography revealed irregular and asymmetric “against-the-rule” astigmatism in both eyes. Anterior segment optical coherence tomography demonstrated numerous central Descemet’s without edema or other corneal structure alterations.

**Conclusion:**

This is the first report of Descemet’s membrane folds in ochronosis. These corneal findings suggest that the accumulation of homogentisic acid in the sclera leads to thickening and stiffness of this region. These alterations could remarkably decrease visual acuity owing to topographic corneal curvature alterations, especially in elderly patients.

## Introduction

Alkaptonuria (AKU) is a rare autosomal recessive metabolic disorder clinically characterized by a triad of dark urine on addition of alkali, ochronotic pigmentation, and degenerative arthritis [1]. Ochronosis is the connective tissue manifestation of AKU caused by deficiency of homogentisate 1,2-dioxygenase activity [1, 2]. This enzyme depletion leads to a complete block within the tyrosine metabolism cascade. Homogentisic acid accumulates and is polymerized into a melanin-like pigment that is ultimately deposited in connective tissue over the years, where it associates with collagen fibers, especially in eyes, skin, bones, tendons, articular cartilages, lungs, valves, and kidneys [2]. Patients with AKU are usually asymptomatic until the fourth decade [3].

Ophthalmic ochronotic changes might be associated with pathologic conditions such as glaucoma, progressive astigmatism, and anterior uveitis [4–8]. Thus, our primary objective is to report for the first time a case of bilateral Descemet’s membrane folds in a patient with ochronosis.

## Case report

An 85-year-old Italian Caucasian male patient presented to our hospital with a substantial bilateral decrease in visual acuity for the past 2 years. He had already been submitted to total replacement of both knees, hip, and cardiac valve in the course of the previous 10 years. Severe motion limitation of spine and shoulders was prevalent. Also, his ears, cartilage, and hands were characteristically pigmented. His family history was negative for alkaptonuric ochronosis. He was treated with analgesic therapy with paracetamol and gabapentin 300 mg × 3/day, vitamin C 1 g daily, dietary restriction of tyrosine and phenylalanine.

**Fig. 1:**
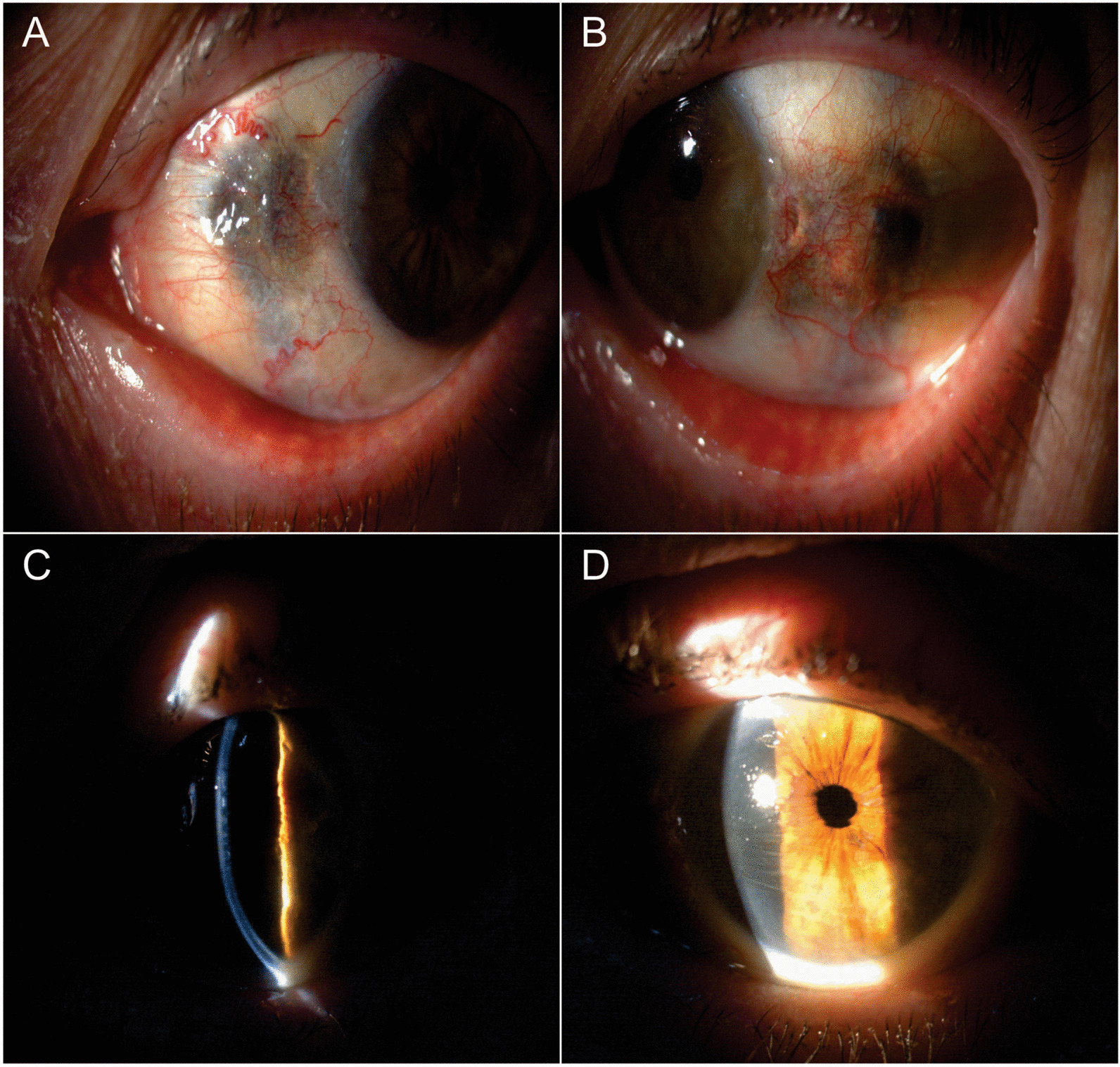
Clinical appearance on silt-lamp biomicroscopy. **A** Dark conjunctival and scleral pigmentation temporally in the right eye. **B** Dark conjunctival and scleral pigmentation temporally in the left eye. **C** Descemet’s membrane folds appearance on cross-section beam. **D** Descemet’s membrane folds appearance on diffuse cross-section beam

The patient presented a corneal topography performed in 2011 that showed relatively regular 5-diopter “against-the-rule” astigmatism in both eyes. Best-corrected visual acuity with spectacles was LogMAR 1.0 (+1.50–6.50×90) and 1.3 (+2.00–7.50×90) for the right and left eye, respectively. On biomicroscopic examination, the patient was pseudophakic (having undergone uneventful phacoemulsification 15 years previously) and presented slightly elevated nodular interpalpebral dark pigmentation in the conjunctiva, sclera, and limbal cornea in both eyes (Fig. 1A, B). Corneal examination showed five horizontal Descemet’s membrane folds in the right eye and three in the left (Fig. 1C, D) with a quiet anterior chamber without any stromal edema or epithelial alteration. Pupils were normal size, reacting to light. Applanation tonometry was 12 mmHg in both eyes, and gonioscopy revealed open angles. Fundus examination with 90D lens showed normal optic disc, macular area, and vessels in both eyes.

**Fig. 2:**
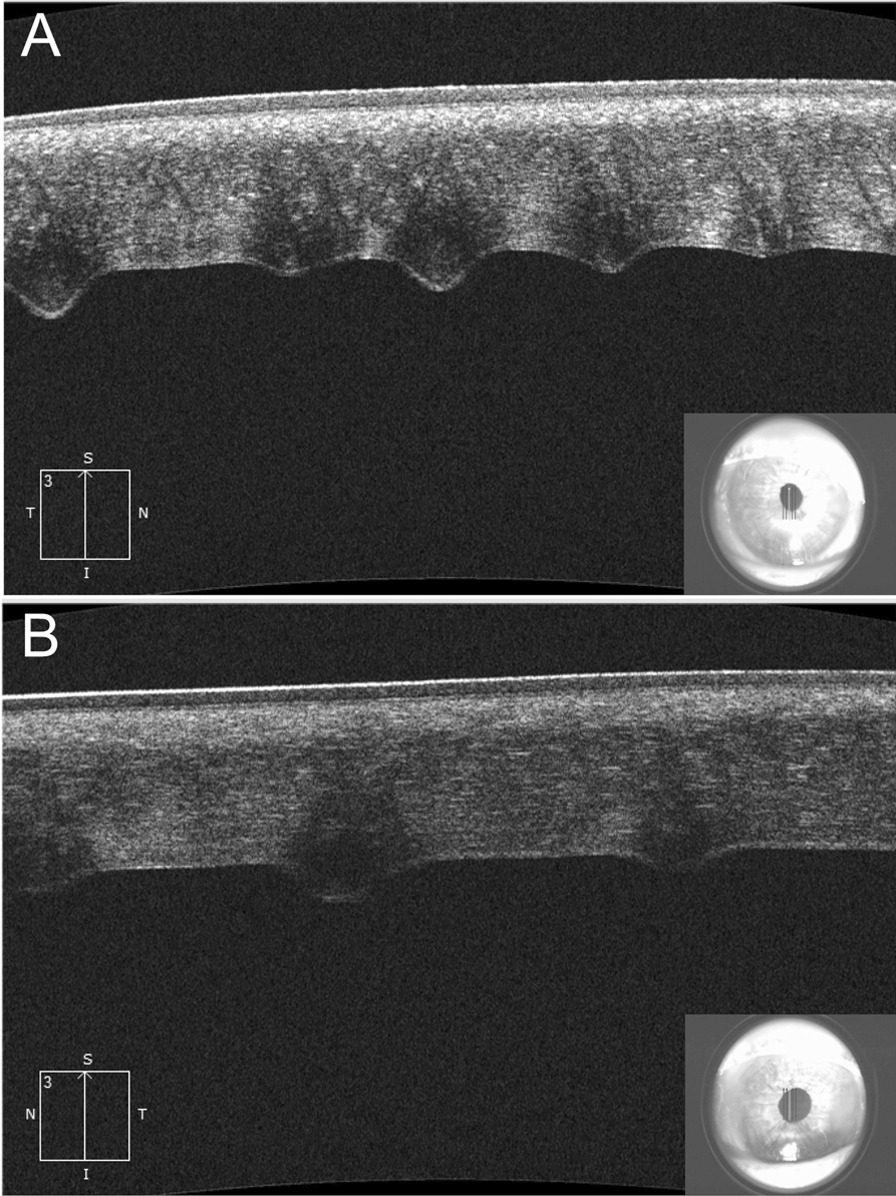
Descemet’s membrane folds on anterior segment optical coherence tomography. **A** Right eye showing five folds with no stromal edema **B** Left eye showing three folds with no stromal edema

**Fig. 3:**
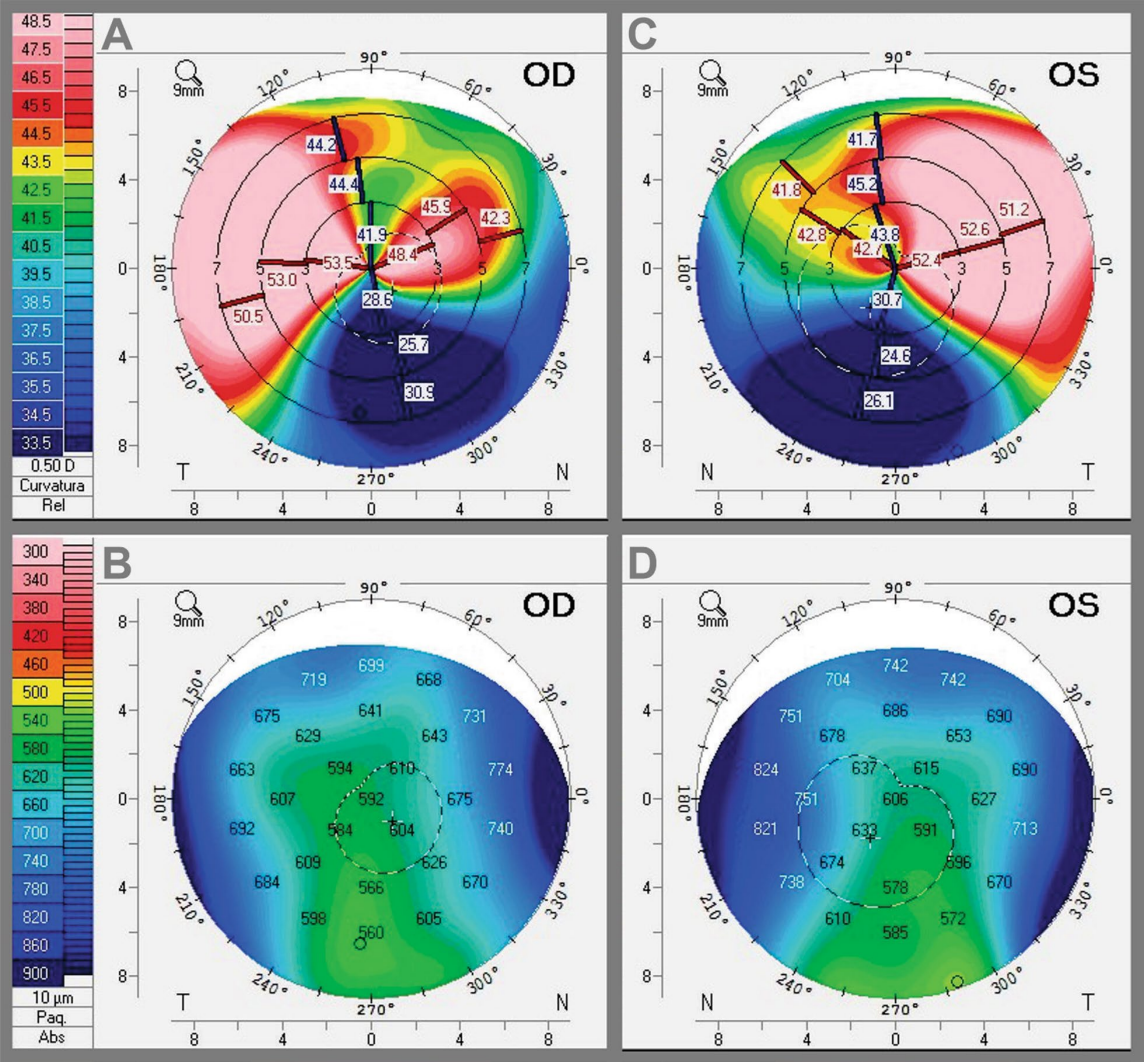
Scheimpflug tomography simulated corneal topography showing anterior sagittal curvature map. **A** Right eye demonstrating 15-diopter asymmetric irregular astigmatism coinciding with the axis of the pigmented lesions. **B** Right eye pachymetry map revealing normal central thickness with inferior displacement of the distribution. **C** Left eye demonstrating 10-diopter asymmetric irregular astigmatism coinciding with the axis of the pigmented lesions. **D** Left eye pachymetry map revealing normal central thickness with inferior displacement of the distribution

The anterior segment analysis mode of the Cirrus HD-OCT 5000 (Carl Zeiss Meditec, Jena, Germany) provided Descemet’s membrane folds details (Fig. 2A, B). Corneal simulated topography in Pentacam HR Scheimpflug tomography (Oculus GmbH, Wetzlar, Germany) revealed irregular and asymmetric astigmatism at 180°, coinciding with the axis of the pigmented lesions in both eyes (Fig. 3A, C). This device showed keratometry (K) readings of 50.00 × 35.00 diopters (D) and 47.00 × 37.00 D in the right and left eye, respectively. Also, the corneal pachymetry map revealed normal central corneal thickness with inferior displacement of the distribution (a thinnest point reading of 578 and 565 μm in the right and left eye, respectively) corresponded to the topographic most flattened area (5 mm from the inferior limbus). The most thickened areas (temporal and nasal) correspond to limbal pigment deposition (Fig. 3B, D).

## Discussion

This case describes the oldest patient with ocular ochronotic findings to be reported in international scientific literature [4]. Progressive, marked “against-the-rule” astigmatism has been reported twice in elderly patients [5, 6]. Both patients were in their seventies when astigmatism began to develop, in both cases being oriented along the same axis as the pigment was found. However, the presence of Descemet’s membrane folds has never been described. Cheskes *et al.* believed that progressive astigmatism was due to corneoscleral thinning after scleral pigment accumulation [6].

Ochronotic pigment in the aortic valve and bone synovium leads to dystrophic calcification, thickening, and stenosis over time [8, 9]. We believe that this is the same process that happens in the sclera. This author has already described a series of 42 patients with osteogenesis imperfecta and thin sclera; however, no corneal folds or irregular astigmatism were observed [10]. The presence of corneal folds suggests that ochronotic accretions, integrated from the sclera into the limbus nasally and temporally, are probably responsible for scleral stiffness in this region. In accordance with this, another study demonstrated no scleral thinning in Anterior Segment Optical Coherence Tomography (AS-OCT) in two patients with ocular ochronosis [11].

In ocular ochronosis, the pigment is typically localized to the sclera, conjunctiva, and limbus. One author described a 25-year-old patient with annular corneal pigmentation in addition to arthropathic changes and skin pigmentation using *in vivo* confocal microscopy [12]. Interestingly, this was not observed in our case. Some authors believe that this intense scleral pigmentation is devoid of cells, suggesting a probable toxic effect of the pigment [13]. The deposition of homogentisic acid polymers occurs around collagen fibrils, altering, thickening, and obscuring their structure, forming plaques and fiber-like structures, followed by necrosis of the fibrocytes. This entire process corroborates our ”scleral stiffening” theory, probably differing due to the duration of the clinical manifestations and severity of the disease.

## Conclusion

This is the first report of Descemet’s membrane folds in ochronosis. These corneal findings, along with other previous studies, suggest that the accumulation of homogentisic acid in the sclera leads to thickening and stiffness of this region. While ochronosis is not known to cause blindness, decreased visual acuity can impact patients’ quality of life in their last decades and hence should be closely observed.

## Data Availability

Not applicable
